# Assessment of γ-H2AX and 53BP1 Foci in Peripheral Blood Lymphocytes to Predict Subclinical Hematotoxicity and Response in Somatostatin Receptor-Targeted Radionuclide Therapy for Advanced Gastroenteropancreatic Neuroendocrine Tumors

**DOI:** 10.3390/cancers13071516

**Published:** 2021-03-25

**Authors:** Thorsten Derlin, Natalia Bogdanova, Fiona Ohlendorf, Dhanya Ramachandran, Rudolf A. Werner, Tobias L. Ross, Hans Christiansen, Frank M. Bengel, Christoph Henkenberens

**Affiliations:** 1 Department of Nuclear Medicine, Hannover Medical School, 30625 Hannover, Germany; Fiona.Ohlendorf@stud.mh-hannover.de (F.O.); Werner.Rudolf@mh-hannover.de (R.A.W.); ross.tobias@mh-hannover.de (T.L.R.); bengel.frank@mh-hannover.de (F.M.B.); 2 Department of Radiation Oncology, Hannover Medical School, 30625 Hannover, Germany; Bogdanova.Natalia@mh-hannover.de (N.B.); christiansen.hans@mh-hannover.de (H.C.); henkenberens.christoph@mh-hannover.de (C.H.); 3 Department of Radiation Oncology, and Gynaecology Research Unit, Hannover Medical School, 30625 Hannover, Germany; Ramachandran.Dhanya@mh-hannover.de

**Keywords:** neuroendocrine tumors, double-strand breaks, γ-H2AX, 53BP1, radionuclide therapy, PRRT

## Abstract

**Simple Summary:**

Assessment of the radiation-induced alterations in peripheral blood lymphocytes is a validated strategy for biological dosimetry and assessment of individual radiosensitivity. However, its clinical significance as a liquid biopsy for predicting hematotoxicity and treatment response when targeting tumor cells with ionizing radiation from a systemically administered radioligand is unexplored. The results from this study revealed that the analysis of radiation-induced foci in peripheral blood lymphocytes may hold potential for predicting both individual subclinical hematotoxicity and tumor response to somatostatin-receptor targeted radioligand therapy in patients with advanced gastroenteropancreatic neuroendocrine tumors. These preliminary data provide the rationale for further studies evaluating analyses-guided treatment intensification in patients at high risk of early progression.

**Abstract:**

Background: We aimed to characterize γ-H2AX and 53BP1 foci formation in patients receiving somatostatin receptor-targeted radioligand therapy, and explored its role for predicting treatment-related hematotoxicity, and treatment response. Methods: A prospective analysis of double-strand break (DSB) markers was performed in 21 patients with advanced gastroenteropancreatic neuroendocrine tumors. γ-H2AX and 53BP1 foci formation were evaluated in peripheral blood lymphocytes (PBLs) at baseline, +1 h and +24 h after administration of 7.4 GBq (^177^Lu)Lu-DOTA-TATE. Hematotoxicity was evaluated using standard hematology. Therapy response was assessed using (^68^Ga)Ga-DOTA-TATE PET/CT before enrollment and after 2 cycles of PRRT according to the volumetric modification of RECIST 1.1. Results: DSB marker kinetics were heterogeneous among patients. Subclinical hematotoxicity was associated with γ-H2AX and 53BP1 foci formation (e.g., change in platelet count vs change in γ-H2AX^+^ cells between baseline and +1 h (*r* = −0.6080; *p* = 0.0045). Patients showing early development of new metastases had less γ-H2AX (*p* = 0.0125) and less 53BP1 foci per cell at +1 h (*p* = 0.0289), and demonstrated a distinct kinetic pattern with an absence of DSB marker decrease at +24 h (γ-H2AX: *p* = 0.0025; 53BP1: *p* = 0.0008). Conclusions: Assessment of γ-H2AX and 53BP1 foci formation in PBLs of patients receiving radioligand therapy may hold promise for predicting subclinical hematotoxicity and early treatment response.

## 1. Introduction

Peptide receptor radionuclide therapy (PRRT) with lutetium-177(^177^Lu)-labelled 1,4,7,10-tetraazacyclododecane‑N,N′,N″,N′′′-tetraacetic acid-d-Phe(1)-Tyr(3)-octreotate (DOTA-TATE) has become a standard treatment option for metastatic somatostatin receptor (SSR)-expressing gastroenteropancreatic neuroendocrine tumors (NETs) [1,2,3], inducing objective radiological responses in up to 18% of patients and significantly improving progression-free survival in advanced midgut NETs with only minor side-effects [1]. However, a substantial number of patients will not respond to (^177^Lu)Lu-DOTA-TATE or progress, which underlines an unmet clinical need for better prediction of the anti-tumor effect of this therapy and for guidance of therapeutic decision-making.

The β-particles emitted by (^177^Lu)Lu-DOTA-TATE induce various types of DNA damage, the most critical being DNA double-strand breaks (DSBs) which can lead to cell death [4,5]. To deal with DSBs, cells activate a rapid and hierarchically coordinated signaling cascade known as DNA damage response (DDR) [5]. One of the earliest responses to the formation of DSBs is the phosphatidylinositol-3-kinase-like-dependent phosphorylation of the H2A histone variant H2AX on serine-139 to form γ-H2AX [5,6], the foci of which form quickly at the sites of DSBs along with a variety of DNA repair factors are localized to the site of the damage [7,8]. The tumor suppressor p53-binding protein 1 (53BP1) also occurs at DSBs, signaling chromatin damage and facilitating ataxia-telangiectasia mutated (ATM)-dependent DSB repair [9,10,11]. Due to the direct response to radiation and the sensitivity of these assays, both markers have emerged for biological dosimetry by measuring DDR signaling [12].

Previous studies have investigated the magnitude and time course of (^177^Lu)Lu-DOTA-TATE therapy-induced DNA damage in peripheral blood lymphocytes (PBLs) of patients with NETs [13,14]. Of note, these studies demonstrated marked DDR heterogeneity among patients, suggesting a potential for stratification beyond mere description of radiation-induced PBL cytotoxicity. Indeed, DDR assessment is increasingly recognized as a functional biomarker of radiosensitivity [15]. Recent studies suggested that DDR analysis in PBLs may have predictive implications for identifying susceptibility to normal-tissue toxicities [16,17]. Importantly, other studies have used DDR foci in PBLs after ex vivo irradiation to detect and characterize DSB repair deficiencies in patients with tumors or leukemia [18], thereby also obtaining information about individual characteristics of DDR.

Based on the aforementioned findings, we hypothesized that an assessment of DDR foci in PBLs of patients undergoing PRRT may hold potential for predicting treatment-related hematotoxicity, and, like other types of liquid biopsy, for predicting the response to therapy, assuming that the somatic DDR in PBLs may at least to some extent still be indicative of individual radiosensitivity of tumors. We tested this hypothesis in a cohort of patients undergoing targeted radioligand therapy for advanced gastroenteropancreatic NETs.

## 2. Results

### 2.1. Validation of DSB Marker Analyses in Immortalized Lymphoblastoid Cells and Healthy Donor PBLs after Irradiation

**Figure 1:**
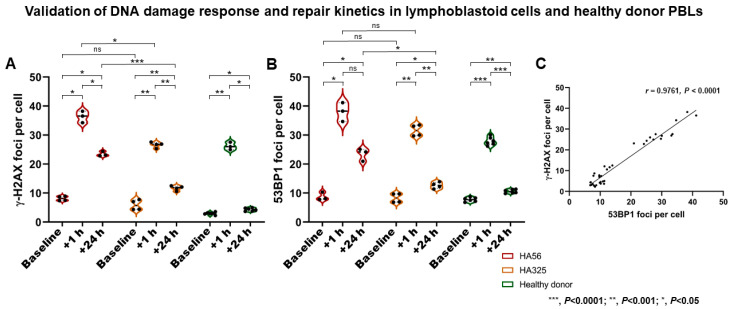
Validation of DNA damage response and repair kinetics analyses. *(***A**,**B**) Following irradiation (2 Gy), immortalized lymphoblastoid cells (LCLs) and healthy donor peripheral blood lymphocytes (PBLs) showed an increase in double-strand break (DSB) marker foci at +1 h (i.e., DNA damage response (DDR)), and a subsequent decrease at +24 h (i.e., repair). The ataxia–telangiectasia (A–T) patient-derived LCL line (HA56) showed more γ-H2AX and 53BP1 foci at +1 h than the wildtype control line (HA325), and a significantly higher number of residual γ-H2AX and 53BP1 foci at +24 h. (**C**) A highly significant correlation between γ-H2AX and 53BP1 foci in LCL samples was found. *** *p* < 0.0001; ** *p* < 0.001; * *p* < 0.05. ns—not significant.

After 2 Gy X-ray treatment, all cell lines showed an increase in DSB marker foci at +1 h, and a subsequent decrease at +24 h. The ataxia–telangiectasia (A–T) patient-derived lymphoblastoid cell line (LCL) (HA56) showed more γ-H2AX and 53BP1 foci at +1 h (*p* = 0.0131 and *p* = 0.1049) than the wildtype control line (HA325) and a significantly higher number of residual γ-H2AX and 53BP1 foci at +24 h (*p* < 0.0001 and *p* = 0.0112, respectively) (Figure 1). There was a highly significant correlation between γ-H2AX and 53BP1 foci in LCLs (*r* = 0.9761 (95% CI, 0.9533 to 0.9878), *p* < 0.0001) and PBLs (*r* = 0.9883 (95% CI, 0.9683 to 0.9957), *p* < 0.0001).

### 2.2. DSB Markers in PBLs Demonstrate Marked Heterogeneity among NET Patients

**Figure 2:**
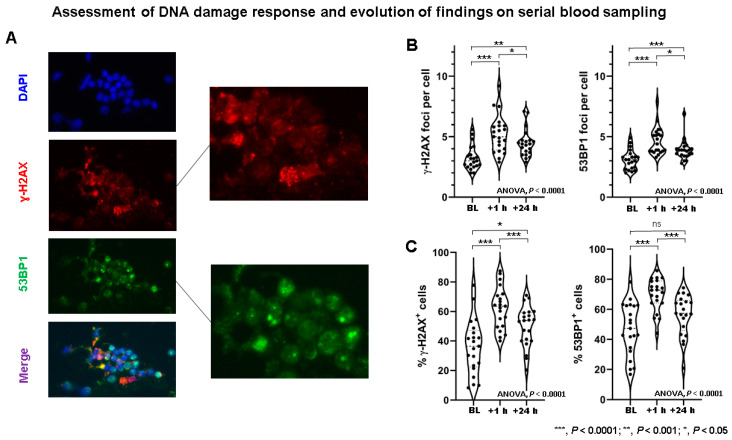
Assessment of DNA damage response in patients receiving peptide receptor radionuclide therapy (PRRT). (**A**) Example of γ-H2AX (red) and 53BP1 (green) foci double immunostaining in PBLs of PRRT patient. DNA was counterstained with DAPI (blue). DSB foci are shown in magnification. (**B**) γ-H2AX foci per cell and 53BP1 foci per cell increased significantly from baseline to +1 h, and then decreased significantly from +1 to +24 h. At +24 h, DDR foci remained significantly elevated compared to baseline. Marked inter-individual heterogeneity in DDR foci per cell can be observed. (**C**) Likewise, the percentage of γ-H2AX^+^ cells and 53BP1^+^ cells increased significantly from baseline to +1 h, and then decreased significantly from +1 to +24 h. At +24 h, DDR foci remained elevated compared to baseline. Marked inter-individual heterogeneity regarding % cells with DDR foci can be observed. BL—baseline; ns—not significant; *** *p* < 0.0001; ** *p* < 0.001; * *p* < 0.05.

**Figure 3:**
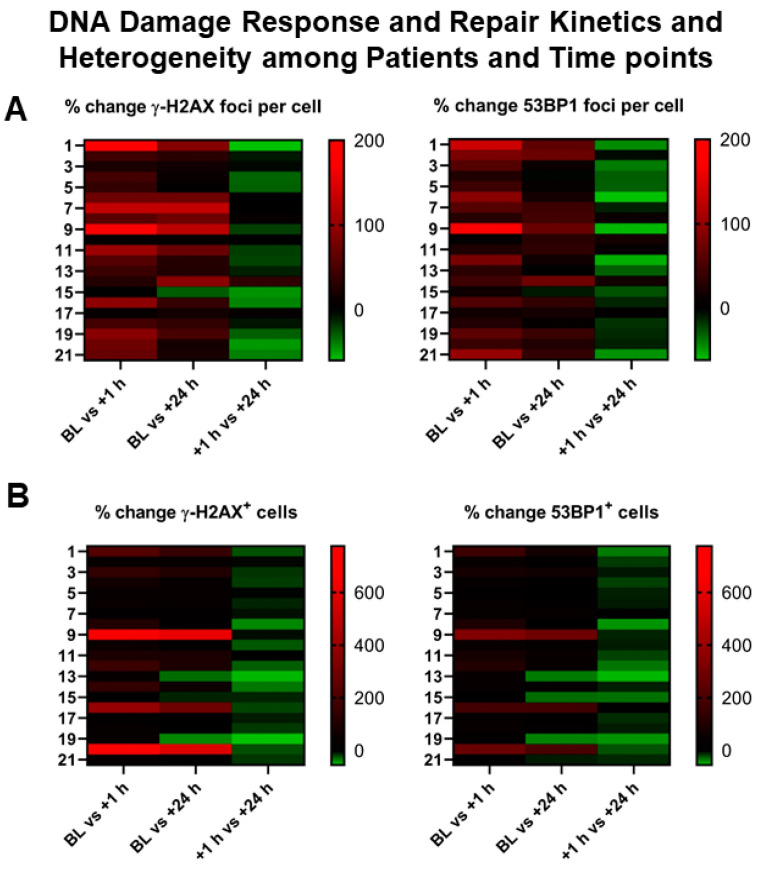
Heterogeneity of DNA damage response in patients receiving PRRT. (**A**,**B**) Graphical illustration (heat map) of inter-individual heterogeneity in DSB markers and their evolution over time, depicted as a heat map ranging from negative % change (green) to high positive % change (red) between different time points (*x*-axis). Left *y*-axis represents patient numbers. Marked inter-individual heterogeneity can be observed in the % change both in induced DDR foci per cell (upper row) and in cells with DDR foci (lower row).

In patients (Table 1), both the number of DSB marker foci per cell and the number of DSB marker-positive cells (Figure 2 and Figure 3) demonstrated considerable inter-individual heterogeneity at all time points (Table S3). A highly significant correlation between γ-H2AX and 53BP1 foci in patient samples was found (*r* = 0.8806; *p* < 0.0001) (Figure S2).

The number of γ-H2AX foci per cell was significantly different between time points (ANOVA, *p* < 0.0001) and increased significantly between BL and +1 h (*p* < 0.0001), then decreased significantly between +1 h and +24 h (*p* = 0.006), but still remained elevated at +24 h compared to baseline (BL) (*p* = 0.0001). Likewise, the number of 53BP1 foci per cell was significantly different between time points (ANOVA, *p* < 0.0001), and increased between BL and +1 h (*p* < 0.0001), then decreased significantly between +1 h and +24 h (*p* = 0.0028), but still remained elevated at +24 h compared to BL (*p* < 0.0001). Similar findings were also found considering the number of DSB marker-positive cells (Table S3). The evolution of DSB maker kinetics as determined by serial blood sampling is summarized in Table S4.

### 2.3. DSB Markers and Hematotoxicity

Following 1 cycle of PRRT, leukocyte count decreased by 13 ± 19% (6.3 × 10^9^/L ± 1.9 × 10^9^/L vs. 5.3 × 10^9^/L ± 1.7 × 10^9^/L; *p* = 0.1053), erythrocyte count decreased by 7 ± 7% (4.4 × 10^12^/L ± 0.6 × 10^12^/L vs. 4.1 × 10^12^/L ± 0.4 × 10^12^/L; *p* = 0.1366), and platelet count decreased by 5 ± 13% (246 × 10^9^/L ± 94 × 10^9^/L vs. 239 × 10^9^/L ± 84 × 10^9^/L; *p* = 0.8120). Post-therapeutic lymphocyte counts were available in 19/21 patients, and declined from 12 ± 0.7 × 10^3^/µL at baseline to 1.0 ± 0.5 × 10^3^/µL; *p* = 0.0012).

**Figure 4:**
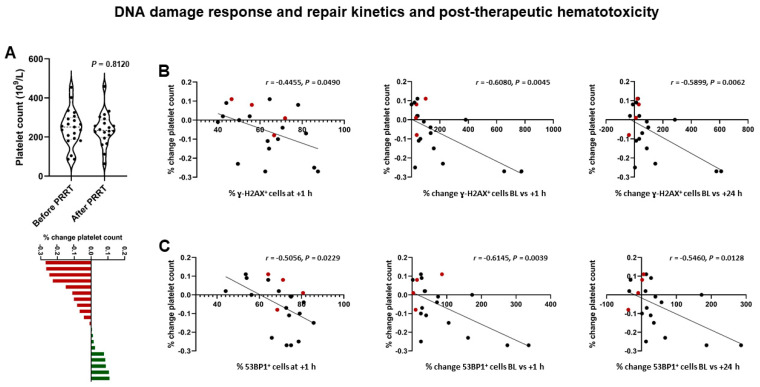
DNA damage response and post-therapeutic subclinical hematotoxicity in patients receiving PRRT. (**A**) Violin plot (upper panel) showing a decrease of −5 ± 13% in platelet count following PRRT on a per-group basis. Waterfall plot (lower panel) showing individual changes in platelet counts, ranging from −27 to +11%. (**B**) Results of correlation analysis. % change in platelet count following PRRT is significantly correlated with the relative number of γ-H2AX^+^ cells at +1 h, and % change of γ-H2AX^+^ cells between baseline and +1 h, and baseline and +24 h. (**C**) Likewise, % change in platelet count following PRRT is significantly correlated with the relative number of 53BP1^+^ cells at +1h, and % change of 53BP1^+^ cells between baseline and +1 h, and baseline and +24 h. Red dots indicate patients with increasing tumor burden. (Platelet count was available in 4/5 patients).

Considering the relationship between DSB markers and post-therapeutic hematotoxicity, DSB-marker kinetics were indicative of subsequent subclinical hematotoxicity (Figure 4), for example: post-therapeutic changes in platelet count correlated with % change in γ-H2AX^+^ cells between baseline and +1 h (*r* = −0.6080 (95% CI, −0.8278 to −0.2264); *p* = 0.045) and % change in 53BP1^+^ cells between baseline and +1 h (*r* = −0.6145 (95% CI, −0.8310 to −0.2362); *p* = 0.0039). Less stringent associations were found for leukocytes (53BP1^+^ cells at +1 h, *r* = −0.4445 (95% CI, −0.7412 to −0.002452); *p* = 0.0496) or erythrocytes (% change γ-H2AX foci per cell baseline vs. +1 h, *r* = 0.4340 (95% CI, −0.01053 to 0.7353); *p* = 0.0559). In patients with increasing tumor burden, platelet count increased by 3 ± 8%.

### 2.4. DSB Markers and Post-Therapeutic Change in Tumor Burden

Following two cycles of PRRT, whole-body tumor burden (SSR-TV) increased by 41 ± 16% (range, 23–64%) in 5 (24%) patients, whereas it remained stable or decreased in the remaining 16 (76%) patients (−25 ± 15% (range, −63 to −0%).

**Figure 5:**
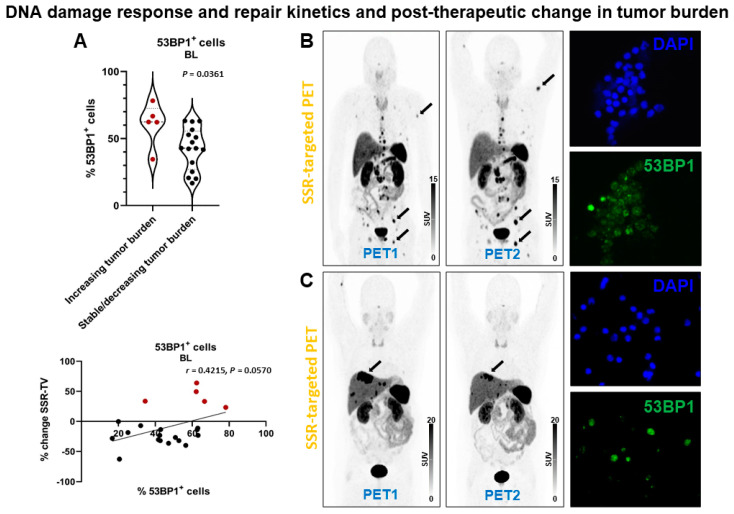
DNA damage response and post-therapeutic change in tumor burden in patients receiving PRRT. (**A**) Violin plot (upper panel) showing % 53BP1^+^ cells at baseline. Patients with stable or decreasing tumor burden had significantly less 53BP1^+^ cells. The change in tumor burden (SSR-TV) demonstrated a borderline significant correlation (lower panel) with the relative number of 53BP1^+^ cells at baseline. Red dots indicate increasing tumor burden. (**B**) Somatostatin receptor (SSR)-targeted maximum-intensity projection PET image before (PET1) and after (PET2) PRRT in a patient with increasing tumor burden (arrows indicate lesions with marked tumor volume increase). This patient had a comparably high relative number of 53BP1^+^ cells at baseline (right lower panel; DAPI nucleic acid staining for comparison, right upper panel). (**C**) Somatostatin receptor (SSR)-targeted maximum-intensity projection PET image before (PET1) and after (PET2) PRRT in a patient with decreasing tumor burden (arrow indicates lesion with marked tumor volume decrease). This patient had a comparably low relative number of 53BP1^+^ cells at baseline (right lower panel; DAPI nucleic acid staining for comparison, right upper panel) (63x/1.6).

Patients with increasing tumor burden had significantly higher % 53BP1^+^ cells at baseline (61 ± 16% (range, 35–78%) vs. 43 ± 16%, (range, 17–63%); *p* = 0.0361) (Figure 5). Consistently, there was a borderline significant association between % change in SSR-TV and % 53BP1^+^ cells at baseline (*r* = 0.4215, *p* = 0.0570) and % change in 53BP1^+^ cells between BL and +24 h (*r* = −0.3866, *p* = 0.0834) in correlation analysis. Linear regression analysis demonstrated a borderline significant association with % 53BP1^+^ cells at baseline (*p* = 0.0570).

### 2.5. DSB Markers Are Associated with Early Development of New Metastases in Patients Receiving Radioligand Therapy

**Figure 6:**
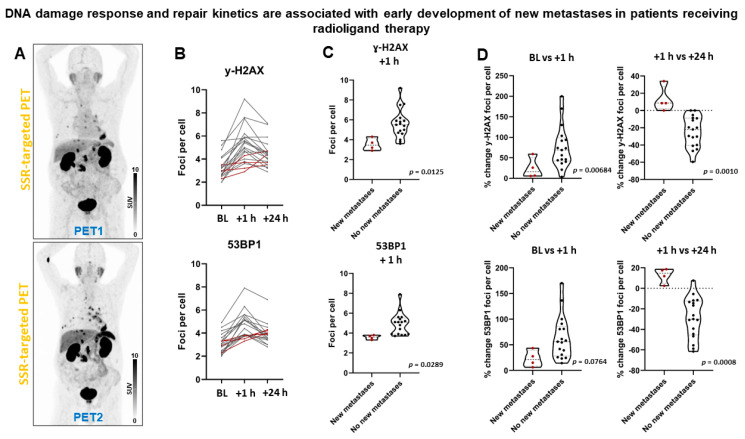
DNA damage response and early development of new metastases in patients receiving PRRT. (**A**) Somatostatin receptor (SSR)-targeted maximum-intensity projection PET image before (PET1) and after (PET2) PRRT in a patient with early development of new thoracic metastases. (**B**) Graphical illustration of individual DSB foci kinetics. Patients with early development of new metastases (dotted red lines) demonstrated a distinct kinetic pattern with absent foci resolution between +1 and +24 h, and low foci increase between baseline and +1 h. (**C**) Patients with early development of new metastases had significantly lower number of DDR foci at +1 h. (**D**) Patients with early development of new metastases did not show resolution of DDR foci between +1 and +24 h.

Following two cycles of PRRT, four (19%) patients progressed (new metastases), whereas all other patients remained stable. All patients with new metastases belonged to the group who had received PRRT before being enrolled, and two (50%) of them developed new metastases in the context of decreasing whole-body tumor burden. Patients with early development of new metastases (Figure 6) did show fewer γ-H2AX foci per cell at +1 h (mean diff., 2.1 ± 0.8; *p* = 0.0125), but not at BL (*p* = 0.4109) or +24 h (*p* = 0.4668). Patients with early development of new metastases did show fewer 53BP1 foci per cell at +1 h (mean diff., 1.3 ± 0.6; *p* = 0.0289), but not at BL (*p* = 0.7081) or +24 h (*p* = 0.6192). 100% of patients with new metastases, but only 2 of 17 patients who did not have new metastases failed to exhibit a reduction in the signal of γ-H2AX foci per cell between +1 and +24 h (*p* = 0.0025). All patients with new metastases, but only 1 of 17 patients without new metastases, showed no reduction in the signal of 53BP1 foci per cell between +1 and +24 h (*p* = 0.0008). The number of γ-H2AX foci increased by 13 ± 15% between +1 and +24 h in patients developing new metastases, whereas it decreased by 25 ± 18% in the remaining patients (*p* = 0.0010). The number of 53BP1 foci increased by 13 ± 7% between +1 and +24 h in patients developing new metastases, whereas it decreased by 29 ± 20% in the remaining patients (*p* = 0.0008). Consistently, logistic regression analysis revealed significant associations with DSB markers (Tables S5 and S6 shows association between hematoxicity and progression). In Kaplan-Meier analysis, progression-free survival (PFS) *(*Figure S3) was significantly shorter in patients with lower number of DDR foci at +1 h (461 days vs not reached; γ-H2AX^+^, *p* = 0.0158; 53BP1, *p* = 0.0241) and absent resolution at +24 h (344 days vs not reached; γ-H2AX^+^, *p* = 0.0058; 53BP1, *p* = 0.0028).

DSB markers at different time points on a per-patient basis are shown in Table S7.

## 3. Discussion

Recently, minimally invasive liquid biopsies have gained increasing attention enabling analysis of tumor components in body fluids such as blood [19]. Liquid biopsies are not limited by constraints of sampling frequency or their incomplete representation of the entire tumor burden [19,20]. In particular, they have demonstrated promise for early detection of therapy resistance [21]. We expanded on this concept and hypothesized that non-tumorous cells may be used to obtain useful information about tumor cell radiosensitivity in the context of systemic radioligand therapy, since the somatic DDR in PBLs may at least to some extent still be indicative of individual radiosensitivity of tumors. To elucidate individual DDR properties, we assessed the foci formation and resolution of two DSB markers (γ-H2AX and 53BP1) before and soon after PRRT. Both represent well-established quantitative read-outs for DNA damage in DSB-causing treatments such as radiation therapy, radionuclide therapy, certain chemotherapies or combination therapies [22,23,24,25,26]. First, we validated our assay by assessing γ-H2AX and 53BP1 foci numbers in irradiated LCLs. It was important to consider that the duration of exposure to radiation is different between external radiation and endoradiotherapy. Radioligand therapy exposes blood cells to radiation for a prolonged time. However, these experiments guided the study design for subsequent PRRT patient samples. In line with the experimental data in LCLs and healthy donor PBLs, we observed similar DDR patterns. Indeed, γ-H2AX and 53BP1 foci disappear with progression of DSB repair [12]. Our observations in PBLs of PRRT patients are consistent with results of previous studies with respect to foci kinetics [13,14], although some variability in absolute number of detected foci is present. Potential explanations may include different experimental protocols, methods of foci quantification (confocal vs. fluorescence microscopy or manual vs. computed), different activity concentrations in the blood and different biokinetics in patients. Of note, we observed marked heterogeneity in both number of DSB foci and number of positive cells at each time point, suggesting a potential for stratification of patients according to individual DDR characteristics. The observed heterogeneity in baseline foci numbers or positive cells may be explained by the characteristics of the patient cohort per se (e.g., age dependence of γ-H2AX foci numbers [27] or previous lines of treatment prior to blood sampling).

Considering treatment-related toxicity, we observed only mild hematotoxicity, which is in line with previous clinical reports [1]. Following 1 cycle of PRRT, neither the leukocyte count (*p* = 0.1053) nor erythrocyte count (*p* = 0.1366) nor platelet count decreased significantly (*p* = 0.8120). However, the lymphocyte count decreased significantly (*p* = 0.0012). Lymphocytes express somatostatin receptors and are particularly radiosensitive. DSB markers were indicative of subsequent subclinical hematotoxicity, in particular in the case of platelets. A decrease in platelet count was paralleled by an increase in DSB markers in PBLs, underlining the value of DSB marker foci in PBLs as a biological dosimetry of radiation-induced damage to the hematopoietic system. Indeed, a previous study has reported a clear correlation between the average number of radiation-induced foci per cell and the absorbed dose to the blood up to 5 h after (^177^Lu)Lu-DOTA-TATE administration [13], enabling the use of DNA-focused damage assays as an in vivo dosimeter. Moreover, in the study by Denoyer et al., the peak number of foci correlated with the absorbed dose to tumor and bone marrow and the extent of PBL reduction [14]. We evaluated a more comprehensive panel of hematological markers beyond PBLs and demonstrated that an assessment of DDR in PBLs may hold potential for estimating the individual toxicity affecting other blood-cell lineages by using a single peripheral cell type to assess the individual radiosensitivity of the total hematopoeitic system, which indeed shares the same hematopoietic stem cells.

Beyond the prediction of subclinical hematotoxicity, we also explored the usefulness of DDR in PBLs for investigating the underlying radiobiology of tumor lesions, similar to other types of liquid biopsy. Indeed, other studies have used DDR foci in PBLs after ex vivo irradiation to detect and characterize DSB repair deficiencies in patients with tumors or leukemias [18]. In this study, patients with an increasing tumor burden following two cycles of PRRT had significantly higher % 53BP1^+^ cells at baseline. Considering that PBLs reflect intrinsic radiosensitivity an increased number of initial DSB-positive cells could indicate genomic instability and a higher likelihood of therapy escape. However, these findings were not robustly confirmed evaluating γ-H2AX or the number of induced foci per cell. Hence, this finding might have limited biological significance and needs to be regarded as needing further confirmation in independent studies.

Of note, patients showing early development of new metastases had fewer DDR foci per cell at +1 h and demonstrated a distinct kinetic pattern with an absence of DSB marker decrease at +24 h in the context of a low baseline level. The predictive value of DDR findings was confirmed in a logistic regression analysis (DSB markers, laboratory values and imaging-derived parameters), and a Kaplan–Meier analysis demonstrated significantly shorter PFS in these patients. Of note, low histological expression of γ-H2AX has been previously shown to be related to poor prognosis in squamous-cell lung cancer [28]. Likewise, the reduced expression of 53BP1 decreased the protein expression of ATM, CHK2, and the phosphorylated products associated with the p53 apoptotic pathway, leading to radiotolerance [29], and low tumor tissue 53BP1 expression was an independent predictor of disease-free survival in patients receiving chemoradiotherapy for rectal cancer [30]. Indeed, 53BP1 contributes to suppression of genomic instability [31]. Therefore, low 53BP1 expression may be associated with genomic instability in this study, providing one potential explanation for therapy escape [32]. In radioiodine therapy, induction of an adaptive response has been observed by a decrease of the cytogenetic damage in peripheral blood lymphocytes after in vitro irradiation as a challenge dose [33], which could also be a potential explanation for low DSB marker levels in pretreated patients. Absent resolution of DDR foci between +1 and +24 h was associated with new metastases and shorter PFS. One might assume that an inability to repair DNA damage leading to accumulation of DDR foci could confer a benefit leading to cell death. However, elucidating the precise mechanisms of non-response to PRRT in NETs would preferably need molecular analyses of histological specimens of metastases [34], which should be considered in future studies. It should be noted that all new metastases occurred in patients who had previously received PRRT before enrollment, leaving unanswered the question of whether the observed predictive DSB pattern would also be useful in patients starting PRRT.

Other groups have suggested PRRT based on individual dosimetry [35], which may become the standard of care and provide potential advantages over fixed-activity empiric PRRT. However, PRRT that uses empiric doses is effective, and fixed standard doses have been used in the NETTER-1 trial leading to approval of the radiopharmaceutical by both the FDA and EMA [1]. Treatment individualization based on individual dosimetry is therefore not an approved form of endoradiotherapy, which raises both legal and ethical issues. Although a personalized PRRT makes it possible to safely increase tumor irradiation in the majority of patients (with acceptable toxicity), its superiority to fixed-activity PRRT has not yet been demonstrated in a randomized controlled trial. However, patients with increasing tumor burden experienced no relevant hematotoxicity in this study, indicating the potential for dose escalation.

Importantly, the same injected activity leads to different absorbed doses in individual patients [13]. Future studies should therefore also evaluate the impact of different absorbed doses to the blood on both DSB marker kinetics in PBLs and on anti-tumor efficacy.

Some limitations should be acknowledged. First, the number of enrolled patients is relatively small, thus limiting statistical power and prohibiting a meaningful multivariate analysis. Nevertheless, the observed associations are significant and would likely become even more compelling in larger cohorts. Furthermore, all previous studies evaluating DDR foci in PRRT included even smaller patient cohorts of 11 to 16 patients [13,14]. However, they evaluated a higher number of blood samples (at different time points) per patient, which provided insight into the temporal pattern of DDR induction and repair. Second, the study population was in part heterogeneous because it included patients who were starting PRRT and some who were receiving later cycles of therapy. However, we did not find any differences in clinical patient characteristics or DSB foci induction and kinetics between these groups (Tables S1 and S2), thereby supporting our decision to pool patients for improved statistical power. Nevertheless, given the relatively small sample size and the heterogeneity of tumors and treatment history, a multivariate analysis would be mandatory for identifying independent predictors of response and progression in the total study population. Third, the single-center design came with inherent limitations, including a potential selection bias representing our local clinical practice in PRRT. Fourth, the numbers of foci per cell depended on the background values of the patients related to intrinsic and extrinsic factors as well as on staining variability [36]. Future studies could evaluate the potential value of subtracting background values for the analysis. For logistical reasons we obtained two blood samples (1 and 24 h) after administration of (^177^Lu)Lu-DOTA-TATE. More samples might have provided an even more comprehensive evaluation of DSB dynamics. Given the inherent limitations, we consider this study to be hypothesis-generating and a stimulus for more expansive efforts.

Finally, dosimetry based on lymphocytes is still under investigation. One recent study on human lymphocytes showed that residual γ-H2AX and 53BP1 foci are very useful markers for biodosimetry [37]. We were interested in the characterization of the DDR in patients receiving PRRT with (^177^Lu)Lu-DOTA-TATE beyond mere biodosimetry and its exploitation for a better understanding of the biology of the response to PRRT. Moreover, our observations were largely consistent with observations in previous studies regarding the foci kinetics in PRRT [13,14]. Our results indicated a potential usefulness of DSB-markers for patient stratification, thus addressing an important question.

## 4. Materials and Methods

### 4.1. Study Cohort

We prospectively enrolled 21 patients (Table 1) referred for PRRT with (^177^Lu)Lu-DOTA-TATE radioligand therapy (RLT) between October 2018 and July 2019 (Figure S1) (64.3 ± 11.6 years; range, 41.0–84.9 years). Eleven patients commenced PRRT, and 10 patients had previously received at least one PRRT cycle before (6 ± 1, range 3–11). All patients suffered from grade 1 (G1) or grade 2 (G2) metastatic nonfunctioning gastroenteropancreatic NETs. Clinical patient characteristics (Table S1), foci induction and DSB marker kinetics were not different between patients commencing PRRT and those having previously received PRRT (Table S2). Therefore (and given the limited statistical power in small subgroups), data were analyzed using the total study population. PRRT was performed according to the joint IAEA, EANM, and SNMMI practical guidance on a compassionate-use basis or in accordance with the Rotterdam protocol [38], with 7.4 GBq (^177^Lu)Lu-DOTA-TATE per cycle every 8 to 16 weeks. Patients discontinued long-acting octreotide for at least 6 weeks before PRRT or short-acting octreotide for at least 24 h before PRRT. A panel of laboratory parameters including standard hematology, serum levels of CgA, alanine aminotransferase (ALT), aspartate aminotransferase (AST), gamma-glutamyl transferase (GGT), lactate dehydrogenase (LDH), and alkaline phosphatase (ALP) was documented for each patient at each cycle. Hematology tests were repeated 2 weeks after each cycle and directly before the next cycle. Hematology levels reflect nadir. (^68^Ga)Ga-DOTA-TATE and (^177^Lu)Lu-DOTA-TATE were administered in compliance with the Declaration of Helsinki and the German Medicinal Products Act, AMG §13.2b. 

### 4.2. Immunocytochemistry Analysis of γ-H2AX and 53BP1 Foci

The reliability of the analysis was confirmed in irradiated lymphoblastoid cells (LCLs) at a dose of 2 Gy from a healthy donor and from an ataxia-teleangiectasia syndrome (A–T) patient, the latter displaying a radiosensitivity phenotype. Lymphoblastoid cells (HA56 (A–T) and HA325 (healthy donor cells)) were established via transformation of B-lymphocytes from peripheral blood by the Epstein–Barr virus [22]. Additionally, for in vitro X-ray irradiation settings, non-immortalised PBLs from one healthy donor were included. The dose of 2 Gy was applied on the cells using a Mevatron MD-2 accelerator (Siemens, Munich, Germany), under conditions equivalent to the usual application of one fraction for radiotherapy and served as a positive control for DSB formation. For each immunocytochemical analysis of PBLs from patients undergoing (^177^Lu)Lu-DOTA-TATE PRRT, 2 Gy irradiated cells were used as positive controls. The results from biological replicates of irradiated cells from at least three independent experiments were analyzed. Both LCLs and patient samples were analyzed before irradiation/(^177^Lu)Lu-DOTA-TATE administration, +1 h after irradiation/(^177^Lu)Lu-DOTA-TATE administration and at +24 h after irradiation/(^177^Lu)Lu-DOTA-TATE administration.

#### 4.2.1. Cell Culture and Isolation of PBLs

Lymphoblastoid cell lines (LCLs) (HA56 and HA325) were cultured in RPMI1640 with 15% fetal calf serum and 500 U/mL penicillin, 0.5 mg/mL streptomycin and 2 mM L-Glutamine. EDTA-blood samples from NET patients were collected before, and at +1 and +24 h after intravenous infusion (5 min duration) of (^177^Lu)Lu-DOTA-TATE. Immediately after sampling, blood samples were placed on ice, weighed and instantly prepared for PBL isolation. PBLs were isolated through Ficoll (GE Healthcare) density-gradient from EDTA blood samples and PBLs from NET patients were frozen in a LCL medium, supplemented with 10% Glycerol at −80 °C until further use, whereas non-immortalised PBLs from one healthy donor were kept in a culture for a maximum of 3 days in a LCL medium for irradiation experiments. All cells were grown at 37 °C in a humidified atmosphere supplemented with 5% CO_2_.

#### 4.2.2. Immunocytochemistry

LCLs and PBLs were centrifuged on cover glasses using a Cytospin ROTANTA 460/460R centrifuge (Hettich, Tuttlingen, Germany). All cells were fixed with 3% (*w*/*v*) PFA, 2% (*w*/*v*) Sucrose in a PBS buffer for 10 min and permeabilised with 0.2% (*v*/*v*) Triton X-100 in PBS. Cells were incubated simultaneously with antibodies against Phospho (S139)-Histone H2AX (Millipore, Burlington, MA, USA) at a ratio of 1:200 and against 53BP1 (Bethyl Laboratories, Montgomery, TX, USA) at a ratio of 1:400 in 2% (*w*/*v*) normal goat serum (Dianova, Hamburg, Germany) for 1 h. After several PBS washing steps, the cells were incubated simultaneously with Alexa Fluor anti-mouse IgG 488 or Alexa Fluor anti-rabbit IgG 546 (Invitrogen, Carlsbad, CA, USA; both at a ratio of 1:250) for 45 min. The DNA was counterstained with DAPI (Invitrogen) and the cells were mounted with ProLong^®^ Gold (Invitrogen).

Immunocytochemistry data evaluation. For quantitative analyses, foci were counted under a Leica DMI6000B microscope using a 63x objective lens at 1.6x magnification (Figure 1). In order to detect all foci in 3D, a manual focus through the whole nucleus was performed. The slides with the patient’s PBLs were coded, so the results were obtained without the knowledge of the patient’s status and clinical details. For each data point, two independent countings by different trained observers were conducted. The counting process was performed independently in several different areas of the slide until at least 50 “positive” cells (with foci) were detected and registered. Every responsive cell (≥1 focus) was included in the evaluation as “positive” since all induced DSBs constitute an important part of the biological response. Cells with apoptotic morphology or cells with intensely stained nucleus were excluded from the counting. For PBLs, monocytes and granulocytes were excluded from the analysis according to morphological criteria.

### 4.3. Preparation of the SSR-Targeting Ligand (^68^Ga)Ga-DOTA-TATE

(^68^Ga)Ga-DOTA-TATE was synthesized by a fully automated, good manufacturing practice (GMP)–compliant procedure using a cassette-based synthesizer (SCINTOMICS GmbH, Fürstenfeldbruck, Germany) connected to a ^68^Ge/^68^Ga-generator (1.2 GBq Ge-68/Ga-68 Generator, itG Isotope Technologies Garching, Garching, Germany) and equipped with a disposable single-use cassette kit (ABX GmbH, Radeberg, Germany). A standardized labeling sequence with 25 μg (17.4 nmol) of unlabeled DOTA-TATE acetate (GMP) (ABX GmbH, Radeberg, Germany) was used. For quality control, (^68^Ga)Ga-DOTA-TATE was analyzed by analytic high-performance liquid chromatography according to the monographs 2462 (Gallium Chloride) and 2482 (Gallium Edotreotide) of the European Pharmacopoeia. Radioanalytic high-performance liquid chromatography was performed on a Varian ProStar high-pressure gradient system equipped with an ultraviolet-visible detector (Varian ProStar 335) and a radiodetector (Berthold LB 3800-20 with LB 6657 probe) using a RP-18 column (Gemini C18 5µ 110A, 250 × 4.6 mm; Phenomenex, Torrance, CA, USA). The eluent had a linear gradient from 100% solvent A (phosphate buffer (pH 2.5)/acetonitrile, 85:15) to 100% solvent B (phosphate buffer (pH 2.5)/acetonitrile, 65:35) over 25 min at a flow of 0.6 mL/min.

### 4.4. PET/CT Acquisition and Image Reconstruction

All studies were acquired using a dedicated PET/CT system (Siemens Biograph mCT 128 Flow; Siemens, Knoxville, TN, USA), equipped with an extended field-of-view PET component, a 128-slice spiral CT component, and a magnetically driven table optimized for continuous scanning. Patients received an intravenous injection of 117 ± 25 MBq of (^68^Ga)Ga-DOTA-TATE. Imaging started with a low-dose, non-enhanced helical CT (120 kV, mA modulated, pitch 1.2, reconstructed axial slice thickness 5.0 mm) for attenuation correction. Whole-body PET images (vertex to mid thighs) were subsequently acquired using continuous bed motion at a speed of 1.3 mm/s for chest and abdomen at 1 h post injection (p.i.) after voiding of the bladder. All studies were reconstructed using Ultra HD^®^, an iterative algorithm combined with time-of-flight and point-spread function information (Siemens Healthcare; 2 iterations, 21 subsets, matrix 200; zoom 1.0; Gaussian filter of 5.0). No contrast material was administered.

### 4.5. Image Analysis and Calculation of Volumetric Parameters

PET/CT images were analyzed using a dedicated workstation equipped with a commercial software package (syngo.via; V10B, Siemens Healthcare), allowing simultaneous and fused review of PET and CT data. All lesions suggestive for metastatic disease were noted, and their localization (e.g., lymph node metastases, bone metastases, hepatic metastases) was recorded. PET images were visually analyzed, and focal uptake of (^68^Ga)Ga-DOTA-TATE higher than background was judged as tissue suspicious of malignancy. The number of detected metastases per patient was recorded. To calculate volumetric parameters, a three-dimensional isocontour volume-of-interest (VOI) including all voxels above 40% of the maximum was created for each lesion using a three-dimensional segmentation and computerized volumetric technique as previously described [39]. This measurement yielded a SSR-derived tumor volume (SSR-TV), similar to the MTV investigated in previous studies [40,41]. Whole-body tumor burden (whole-body SSR-TV) was determined by summing up SSR-TV measurements of all individual lesions in each patient.

### 4.6. GMP-Compliant Preparation of the SSR-Targeting Ligand (^177^Lu)Lu-DOTA-TATE

Lutetium-177 was purchased from itg (Isotope Technologies Garching GmbH, Germany) as GMP-certified ^177^Lu-LuCl_3_ in 0.04M HCl-solution (EndolucinBeta^TM^, 40 GBq/mL) in no carrier added quality. The precursor DOTA-TATE) was achieved from ABX (Germany) in GMP quality. The radiosynthesis was performed on a Gaia/Luna GMP automated radiosynthesizer (Elysia-raytest GmbH, Straubenhardt, Germany) using a sterile, single-use cassette and reagent kit (ABX, Germany). Per patient dose, 150 µg DOTA-TATE precursor was dissolved in 800 µL buffer solution (gentisic acid/sodium ascorbate/HCl). Between 7.0 and 9.0 GBq (^177^Lu)LuCl_3_ per patient was provided in the sterile, rubber-sealed delivery vial (10 mL), which served as reaction vessel in the automated process. The ^177^Lu-labelling step was conducted at 95 °C for 30 min. The product solution was transferred into a product vial via a sterile filter and diluted by 10–15 mL 0.9% NaCl. RadioHPLC as primary quality control was performed on a Merck HPLC system equipped with two L-7100 pumps, a L-7200 autosampler, a L-7400 UV/Vis detector, a D-7000 interface d-line and a GABI radiodetector (Elysia-raytest, Straubenhardt, Germany), and a Gemini C18, 5 µm, 100 Å column (250 × 4.6 mm) (Phenomenex, Aschaffenburg, Germany). As eluent phosphate buffer (pH 2) and acetonitrile was used in a gradient system at a flow of 0.6 mL/min. Production batches were further tested for pH, sterility, endotoxins and radionuclide purity (gamma spectroscopy). (^177^Lu)Lu-DOTA-TATE was always of flawless quality with radiochemical purity of ≥97% and a peptide content of 18.5–19.5 µg/GBq.

### 4.7. Clinical Endpoints

Treatment response was evaluated using (^68^Ga)Ga-DOTA-TATE PET according to RECIST 1.1 criteria adapted to volumetric measurements [39,42,43]. To this end, the volumetry of all tumor lesions was performed as previously described [39], and whole-body volumetric parameters were compared between scans. Patients underwent a baseline PET before first PRRT in case of new patients, followed by a PET/CT after 2 cycles (about 6–8 weeks after the second cycle). In patients having previously received PRRT, PET/CT was performed after every two cycles. A progressive disease was defined as the appearance of new lesions or an increase in tumor burden greater or equal to 73% [42,43]. We separately analyzed both the relationship between DSB markers and the change in SSR-TV and between DSB markers and the early occurrence of new metastases.

### 4.8. Statistical Analysis

Categorical variables are presented with absolute and relative frequencies. Continuous variables are expressed as mean ± standard deviation (SD) and range. The D’Agostino–Pearson omnibus normality test was performed to verify normal distribution of data. DSB marker foci per cell from LCLs and healthy donor PBLs were compared using a mixed effects model with the Geisser–Greenhouse correction and Tukey’s multiple comparison test, with individual variances computed for each comparison. DSB marker foci per cell and % cells at different time points in patients were compared using a repeated-measures analysis of variance (ANOVA) with the Geisser–Greenhouse correction and Tukey’s multiple comparison test, with individual variances computed for each comparison. DSB marker heterogeneity was visualized using heat maps. Pearson correlation was used to explore the association of DSB markers and hematotoxicity and the change in tumor burden. A student’s *t*-test was used to compare differences in DSB markers between groups. Fisher’s exact test was used to compare categorical variables between groups. A paired *t*-test was used to compare pretherapeutic and post-therapeutic lymphocyte levels. Fisher’s exact test was used to evaluate the relationship between occurrence of new metastases and DSB kinetic patterns. Survival analysis (PFS) was performed using the Kaplan–Meier method, and group data were compared using the Gehan–Breslow–Wilcoxon test. Cut-offs were derived using receiver operating characteristic (ROC) analyses. Clinical patient characteristics (Table S1), foci induction and DSB marker kinetics were not different between patients commencing PRRT and those having previously received PRRT (Table S2). Therefore (and given the limited statistical power in small subgroups), data were analyzed using the total study population. Statistical significance was established for *p* values of <0.05. Statistical analysis was performed using GraphPad Prism (version 8.3 for Windows; Graphpad Software, San Diego, CA, USA).

## 5. Conclusions

DSB foci induction and kinetics in PBLs of patients receiving PRRT demonstrated marked heterogeneity, indicating a potential for patient stratification. Beyond being indicative of subclinical hematotoxicity, these parameters may hold promise for the prediction of treatment response and risk of progression by elucidating individual DNA damage response properties; however, these data should be considered preliminary at this point. This study provided a rationale for larger prospective studies further establishing the value of DDR analyses in PRRT patients and evaluating DDR analyses-guided treatment intensification in patients at high risk of early progression.

## Figures and Tables

**Table 1:** Characteristics of study population (*n* = 21).

Parameter	Value
Gender *no*. (%)	
Male	10 (48%)
Female	11 (52%)
Age (years)	
Mean ± SD	64.3 ± 11.6
Range	41.0–84.9
Body–mass index (kg/m²)	
Mean ± SD	26.9 ± 6.0
Range	18.1–42.8
Karnofsky performance status no. (%)	
≤70%	1 (5%)
>70%	20 (95%)
Primary tumor site no. (%)	
Pancreas	6 (29%)
Small intestine	14 (67%)
Ileum	8 (38%)
Jejunum	3 (14%)
Small intestine, not otherwise specified	3 (14%)
Rectum	1 (5%)
Site of metastases no. (%)	
Liver	20 (95%)
Lymph nodes	13 (62%)
Bone	8 (38%)
Ki-67 index no. (%)	
≤2% (G1)	7 (33%)
3–20% (G2)	14 (67%)
Chromogranin A (µg/l)	
Mean ± SD	390 ± 520
Range	24–2179
Krenning score no. (%)	
Grade 2	0 (0%)
Grade 3	6 (29%)
Grade 4	15 (71%)
Previous therapies no. (%)	
Surgery	14 (67%)
Chemotherapy	4 (19%)
Everolimus	5 (24%)

G—grade; No.—number; SD—standard deviation.

## Data Availability

The data are not publicly available because, due to the European regulations regarding data protection, we cannot make data available online or send it. However, all data are available for revision on-site.

## Supplementary Materials

The following are available online at https://www.mdpi.com/2072-6694/13/7/1516/s1, Figure S1, Flow chart of study population; Figure S2, Correlation between γ-H2AX and 53BP1 foci in patient samples; Figure S3, DNA damage response and repair kinetics and progression-free survival (PFS) in patients receiving PRRT; Table S1, Characteristics of study population (*n* = 21); Table S2, Number of double-strand break (DSB) marker foci per cell and DSB marker-positive cells at different time points in patients receiving peptide receptor radionuclide therapy (PRRT); Table S3: Number of double-strand break (DSB) marker foci per cell and DSB marker-positive cells at different time points in patients receiving peptide receptor radionuclide therapy (PRRT); Table S4, Evolution of double-strand break (DSB) marker foci per cell and DSB marker-positive cells on serial blood sampling in patients receiving peptide receptor radionuclide therapy (PRRT); Table S5, Predictors of new metastases after 2 cycles of PRRT in the study population (*n* = 21); Table S6, Early percentage change in platelet count, change in tumor burden and appearance of new metastases after 2 cycles of PRRT in the study population (*n* = 21); Table S7, Number of DSB foci per cell and % of DSB-marker positive cells at different time-points on a per-patient basis.
