# Supplementary material for: Assessment of γ-H2AX and 53BP1 Foci in Peripheral Blood Lymphocytes to Predict Subclinical Hematotoxicity and Response in Somatostatin Receptor-Targeted Radionuclide Therapy for Advanced Gastroenteropancreatic Neuroendocrine Tumors
Source: Cancers (Basel). 2021 Mar 25;13(7):1516. doi: 10.3390/cancers13071516 (PMC8036952; doi:10.3390/cancers13071516)
Supplement: Supplementary file 1 [file cancers-13-01516-s001.pdf]

Assessment of γ-H2AX and 53BP1 Foci in Peripheral Blood Lymphocytes to Predict Subclinical Hematotoxicity and Response in Somatostatin Receptor-Targeted Radionuclide Therapy for Advanced Gastroenteropancreatic Neuroendocrine Tumors

Table S1. Characteristics of study population (*n* = 21).

Parameter	Patients commencing PRRT	Patients already having received PRRT	*P* value
Number	11 (52%)	10 (48%)	
Gender no. (%)			0.3949
Male	4 (36%)	6 (60%)	
Female	7 (19%)	4 (40%)	
Age (years)			0.2011
Mean ± SD	61.2 ± 10.1	67.78 ± 12.7	
Range	41.0 to 72.8	50.6 to 84.9	
Body-mass index (kg/m²)			0.7138
Mean ± SD	26.4 ± 7.5	27.4 ± 4.2	
Range	18.1 to 42.8	22.5 to 34.3	
Karnofsky performance status no. (%)			1.000
≤ 70 %	1 (9%)	0 (0%)	
> 70 %	10 (91%)	10 (100%)	
Primary tumor site no. (%)			
Pancreas	2 (18%)	4 (40%)	0.3615
Small intestine	9 (82%)	5 (50%)	0.1827
Ileum	7 (64%)	1 (10%)	
Jejunum	1 (9%)	2 (20%)	
Small intestine, not otherwise specified	1 (9%)	2 (20%)	
Rectum	0 (0%)	1 (10%)	0.4762

Site of metastases no. (%)			
Liver	11 (100%)	9 (90%)	0.4762
Lymph nodes	7 (64%)	6 (60%)	1.000
Bone	5 (45%)	3 (30%)	0.6594
Other	8 (73%)	3 (30%)	0.0861
Ki-67 index no. (%)			1.000
≤ 2 % (G1)	4 (36%)	3 (30%)	
3-20 % (G2)	7 (19%)	7 (70%)	
Chromogranin A (μg/l)			0.4493
Mean ± SD	475 ± 627	298 ± 380	
Range	44 to 2179	24 to 1234	
Krenning score no. (%)			
Grade 2	0 (0%)	0 (0%)	1.000
Grade 3	4 (36%)	2 (20%)	0.6351
Grade 4	7 (64%)	8 (80%)	0.6351
Previous therapies no. (%)			
Surgery	8 (73%)	6 (60%)	0.6594
Chemotherapy	4 (36%)	0 (0%)	0.0902
Everolimus	3 (27%)	2 (20%)	1.000

G – grade; No. – number; SD – standard deviation

Table S2 Number of double-strand break (DSB) marker foci per cell and DSB marker-positive cells at different time points in patients receiving peptide receptor radionuclide therapy (PRRT).

Parameter	Patients commencing PRRT			Patients already having received PRRT					
	Time point			Time point					
	**BL**	**+1h**	**+24h**	**BL**	***P* value**	**+1h**	***P* value**	**+24h**	***P* value**
No. of γ-H2AX foci per cell									

Mean±SD	3.3 ± 1.0	5.6 ± 1.8	4.6 ± 1.5	3.2 ± 1.0	0.9427	4.8 ± 1.2	0.2558	4.3 ± 0.5	0.6172
Range	2.0 to 5.2	3.6 to 9.2	2.9 to 7.1	2.0 to 5.6		2.9 to 6.2		3.5 to 5.3	
% γ-H2AX⁺ cells									
Mean±SD	39% ± 17%	65% ± 14%	54% ± 12%	33% ±21%	0.4209	59% ± 14%	0.2978	46% ± 14%	0.2088
Range	10% to 69%	44% to 88%	30% to 71%	8% to 78%		40% to 82%		20% to 69%	
No. of 53BP1 foci per cell									
Mean±SD	3.1 ± 1.0	4.9 ± 1.3	3.9 ± 1.2	3.1 ± 0.7	0.8362	4.4 ± 0.9	0.3319	4.0 ± 0.3	0.8805
Range	2.1 to 4.9	3.8 to 7.9	2.8 to 6.9	2.0 to 4.2		3.3 to 5.6		3.4 to 4.5	
% 53BP1⁺ cells									
Mean±SD	51% ± 18%	71% ± 10%	58% ± 12%	43% ± 17%	0.3095	69% ± 12%	0.7432	54% ± 16%	0.4715
Range	20% to 78%	54% to 81%	39% to 74%	17% to 67%		44% to 86%		21% to 70%	

*53BP1 - tumor suppressor p53-binding protein 1; BL – baseline; No. – number; SD – standard deviation; γ-*H2AX *- phosphorylated form of H2A histone family member X*

Table S3. Number of double-strand break (DSB) marker foci per cell and DSB marker-positive cells at different time points in patients receiving peptide receptor radionuclide therapy (PRRT).

Parameter	Time point		
	BL	+1h	+24h
No. of γ-H2AX foci per cell			
Mean±SD	3.3 ± 1.0	5.2 ± 1.6	4.5 ± 1.1
Range	2.0 to 5.6	2.9 to 9.2	2.9 to 7.1
% γ-H2AX⁺ cells			
Mean±SD	36% ± 19%	62% ± 14%	50% ± 14%
Range	8% to 78%	40% to 88%	20% to 71%
No. of 53BP1 foci per cell			
Mean±SD	3.1 ± 0.8	4.7 ± 1.1	3.9 ± 0.8

Range	2.0 to 4.9	3.3 to 7.9	2.8 to 6.9
% 53BP1⁺ cells			
Mean±SD	47% ± 17%	70% ± 11%	56% ± 14%
Range	17% to 78%	44% to 86%	21% to 74%

53BP1 - tumor suppressor p53-binding protein 1; BL – baseline; No. – number; SD – standard deviation; γ-H2AX - phosphorylated form of H2A histone family member X

Table S4 Evolution of double-strand break (DSB) marker foci per cell and DSB marker-positive cells on serial blood sampling in patients receiving peptide receptor radionuclide therapy (PRRT).

	Mean difference	95% CI of difference	*P* value
No. of γ-H2AX foci per cell			
BL vs +1h	+1.9	+1.2 to 2.7	<0.0001
BL vs +24h	+1.2	+0.6 to +1.8	0.0001
+1h vs +24h	-0.8	-1.3 to -0.2	0.0060
% γ-H2AX⁺ cells			
BL vs +1h	+26%	+16% to +36%	<0.0001
BL vs +24h	+14%	+4% to +24%	0.0069
+1h vs +24h	-13%	-18% to -7%	<0.0001
No. of 53BP1 foci per cell			
BL vs +1h	+1.6	+1.0 to +2.1	<0.0001
BL vs +24h	+0.8	+0.5 to +1.2	<0.0001
+1h vs +24h	-0.8	-1.2 to -0.3	0.0028

% 53BP1^{+} cells			
BL vs +1h	+23%	+14% to +32%	<0.0001
BL vs +24h	+9%	+0% to +19%	0.0646
+1h vs +24h	-14%	-19% to -9%	<0.0001

53BP1 - tumor suppressor p53-binding protein 1; BL – baseline; No. – number; SD – standard deviation; γ-H2AX *- phosphorylated form of H2A histone family member X*

Table S5 Predictors of new metastases after 2 cycles of PRRT in the study population (*n*=21).

Parameter	Odds ratio	95% CI	*P* value
<u>DSB markers</u>			
No. of γ-H2AX foci per cell			
BL	0.5377	0.08255 to 1.806	0.3524
+1h	0.05858	0.0005325 to 0.4587	**0.0010**
+24h	0.6160	0.1270 to 1.786	0.4118
% change γ-H2AX foci per cell			
BL to +1h	0.9514	0.8863 to 0.9935	**0.0167**
BL to +24h	1.002	0.9729 to 1.030	0.8693
+1h to +24h		Perfect separation	
No. of 53BP1 foci per cell			
BL	0.7430	0.1353 to 2.977	0.6857
+1h	$4.142e^{-008}$	$3.091e^{-023}$ to 0.06446	**0.0003**
+24h	1.364	0.3278 to 4.851	0.6158
% change 53BP1 foci per cell			
BL to +1h	0.9295	0.8243 to 0.9905	**0.0149**
BL to +24h	1.025	0.9793 to 1.079	0.2900
+1h to +24h	1.298	1.080 to 2.307	**<0.0001**
No. of γ-H2AX^{+} cells			
BL	1.008	0.9468 to 1.073	0.7833
+1h	1.004	0.9237 to 1.091	0.9155
+24h	0.9732	0.8928 to 1.057	0.5085
% change γ-H2AX^{+} cells			
BL to +1h	0.9964	0.9786 to 1.003	0.3708
BL to +24h	0.9916	0.9622 to 1.003	0.2162

+1h to +24h	0.9624	0.8912 to 1.034	0.2824
No. of 53BP1^{+} cells			
BL	1.027	0.9612 to 1.113	0.4432
+1h	1.041	0.9354 to 1.204	0.4892
+24h	1.026	0.9443 to 1.150	0.5691
% change 53BP1^{+} cells			
BL to +1h	0.9926	0.9624 to 1.007	0.3766
BL to +24h	0.9914	0.9589 to 1.008	0.3716
+1h to +24h	1.003	0.9319 to 1.097	0.9297
<u>Laboratory values</u>			
Leucocyte count	0.4220	0.1179 to 0.9422	**0.0326**
Erythrocyte count	0.01582	8.592^{e-005} to 0.2756	**0.0015**
Thrombocyte count	0.9958	0.9821 to 1.008	0.5035
Aspartate transaminase (AST)	0.9563	0.8166 to 1.038	0.3524
Alanine transaminase (ALT)	0.9111	0.7106 to 1.010	0.1412
Lactate dehydrogenase (LDH)	0.9169	0.8006 to 0.9813	**0.0041**
Alkaline phosphatase (ALP)	1.000	0.9871 to 1.011	0.9508
Estimated glomerular filtration rate (eGFR)	1.010	0.9378 to 1.093	0.7907
Chromogranin-A (CgA)	0.9965	0.9866 to 1.001	0.1350
<u>Imaging-derived markers</u>			
SSR-TV	1.001	0.9826 to 1.016	0.8629
TL-SSR	1.000	0.9998 to 1.001	0.3311
T/S ratio	1.038	0.5957 to 1.568	0.8659
Maximum SUV_{max}	1.020	0.9646 to 1.081	0.4761

Table S6 Early percentage change in platelet count, progression in tumor burden and appearance of new metastases after 2 cycles of PRRT in the study population (*n*=21).

Patient no.	% change platelet count	Response	Progression TV	New metastases
1	-23%	SD	n	n
2	+2%	SD	n	n
3	-4%	SD	n	n
4	-10%	SD	n	n

5	+8%	SD	y	n
6	+11%	PD	n	y
7	+9%	SD	n	n
8	+11%	PD	y	y
9	-27%	SD	n	n
10	-11%	SD	n	n
11	-1%	SD	n	n
12	-15%	SD	n	n
13	+2%	SD	n	n
14	-7%	PD	n	y
15	+8%	SD	n	n
16	0%	SD	n	n
17	n.a.	PD	y	y
18	-25%	SD	n	n
19	-8%	SD	y	n
20	-27%	SD	n	n
21	+1%	SD	y	n

n – no; no – number; PD – progressive disease; SD – stable disease; TV – tumor volume; y - yes

Table S7 Number of DSB foci per cell and % of DSB-marker positive cells at different time-points on a per-patient basis

Patient no.	No. of y-H2AX foci per cell			No. of 53BP1 foci per cell			% of y-H2AX⁺ cells			% of 53BP1⁺ cells		
	BL	+1h	+24h	BL	+1h	+24h	BL	+1h	+24h	BL	+1h	+24h
1	2,5	7,5	4,7	2,2	5,2	3,6	15,4	49,5	38	25,2	65,9	42,3
2	2,5	3,6	3,3	2,1	3,8	3,6	39,6	55,1	53,2	50,9	69,2	56,8
3	3,3	4	3,8	2,5	3,9	2,8	30,2	70,7	59,6	47,2	81	74,3
4	2,6	3,8	2,9	3,1	3,9	3	42,2	68,5	56,3	63,2	79,1	64,1
5	4,1	5,6	4,3	3,6	5,1	3,9	41,6	56,1	54,5	62,4	71,2	63,6
6	3,4	5,9	5,9	3,3	6,3	3,9	45,5	64,6	57,1	42,7	53,6	48,5
7	2	4,6	4,6	2,4	3,8	3,4	36,4	44	41,2	42,4	54	54
8	2,7	4,3	4,7	2,9	3,7	4,2	23,3	46,5	28,2	34,5	64,2	36,6
9	2	5,4	4,5	2	5,4	3,4	8,3	62,5	59	16,7	72,9	64,4
10	3,5	3,7	3,7	3,3	3,5	4,3	42,1	63,6	47,2	52,8	74,1	66,7
11	2,7	5,5	4,6	3	3,7	4	21,4	40	40	42,9	75	60
12	3,2	5	4,1	3,1	5,6	3,6	25	64,3	46,8	41,7	85,7	56,5
13	4,2	5,9	5,3	3,9	5,1	3,9	29,5	42,4	20,3	32,1	44,1	20,8
14	2,3	2,9	4,4	2,3	3,3	4	34,8	81,8	53,8	56,5	72,7	65
15	5,6	5,8	4	4,2	4,8	3,8	77,8	78,1	69,1	63	64,2	43,2
16	3,2	6,2	4,4	3,3	5,1	4,5	10,4	50,1	40	20,6	56,3	56,1
17	3	3,2	3,5	3,3	3,8	3,9	53,3	60,2	54,2	66,7	81	69,6
18	5,2	7,6	7	4,5	5,6	4,8	68,5	85,7	70,9	62,7	78,6	70,8
19	4,8	9,2	7,1	4,9	7,9	6,9	48,7	66,7	30	62,2	68,5	38,8
20	2,7	4,7	3,2	2,8	3,9	3,5	10	87,5	67,7	20	75	57,3
21	2,9	4,9	3,5	2,2	4,4	3	54,5	71,9	60	78,2	80,7	70,8

BL – baseline value; no. – number

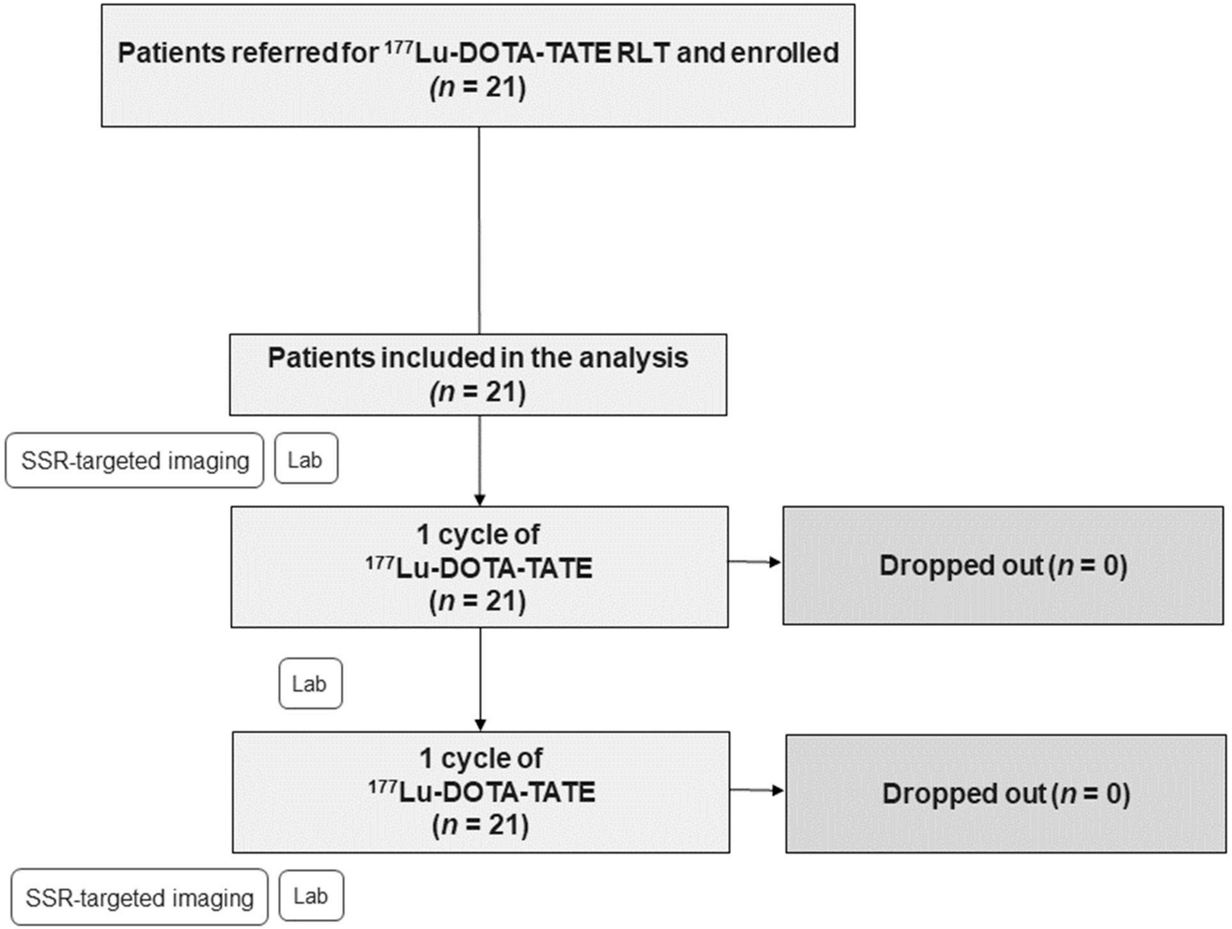

Figure S1. *Flow chart of study population.* 21 patients were included in the analysis. Each patient underwent a baseline ^{68}Ga-DOTA-TATE PET/CT scan before commencing PRRT (*n* = 11) or in the course of PPRT (*n* = 10). Following two cycles of PRRT, a follow-up ^{68}Ga-DOTA-TATE PET/CT was obtained for assessment of change in tumor burden and occurrence of new metastases. A panel of clinical laboratory values including standard hematology was assessed before each cycle.

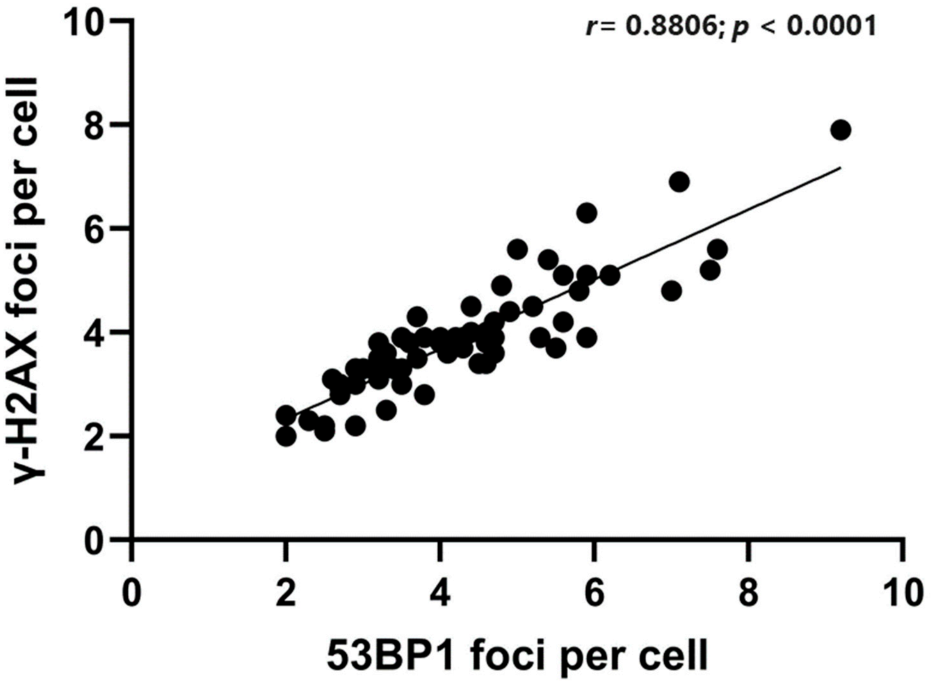

Figure S2 *Correlation between γ-H2AX and 53BP1 foci in patient samples.*

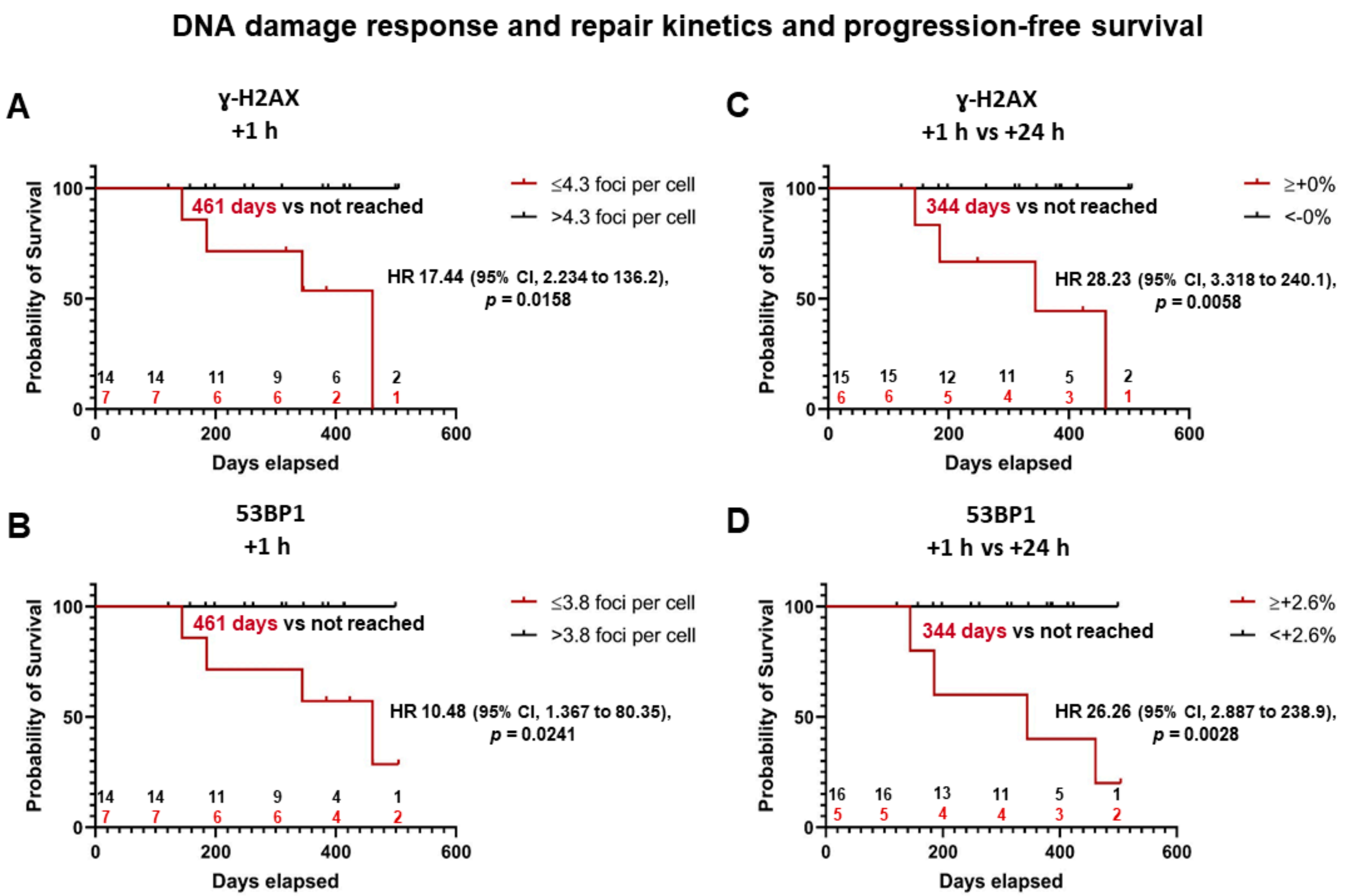

Figure S3 *DNA damage response and repair kinetics and progression-free survival (PFS) in patients receiving PRRT.* **A**, **B** PFS was significantly shorter in patients with a lower number of DDR foci at +1h (461 days vs not reached; γ-H2AX+, *P* = 0.0158; 53BP1, *P* = 0.0241). **C**, **D** PFS was significantly shorter in patients with absent resolution of DDR foci between +1h and +24h (344 days vs not reached; γ-H2AX+, *P* = 0.0058; 53BP1, *P* = 0.0028).
